# Clozapine-Induced Phototoxicity: An Unusual Side Effect of Atypical Antipsychotics

**DOI:** 10.1155/2018/9242515

**Published:** 2018-06-25

**Authors:** S. Al-Aojan, A. Al-Khalifah

**Affiliations:** Prince Sultan Military Medical City, Riyadh, Saudi Arabia

## Abstract

**Introduction:**

Clozapine is a second-generation antipsychotic used for treatment-refractory schizophrenia. Photosensitivity is a major concern when prescribing typical antipsychotics, while atypical antipsychotics are thought to be less photosensitive.

**Case Report:**

We report a 27-year-old military personnel who developed a phototoxic drug reaction after using clozapine for schizophrenia.

**Conclusion:**

Recognition of rare but possible ability of atypical antipsychotics to cause photosensitivity is pertinent to patients care and proper counseling.

## 1. Introduction

Clozapine is a second-generation antipsychotic. It is the standard of care for treatment-refractory schizophrenia and for reducing the risk of suicidal behaviors in schizophrenia and schizoaffective disorder [1]. It is associated with a variety of side effects including agranulocytosis, myocarditis, seizures, weight gain, constipation, and others [2]. While photosensitivity is more commonly seen with first-generation antipsychotics [3, 4], this is the second reported case of clozapine-induced photosensitivity in the literature so far.

## 2. Case Report

**Figure 1:**
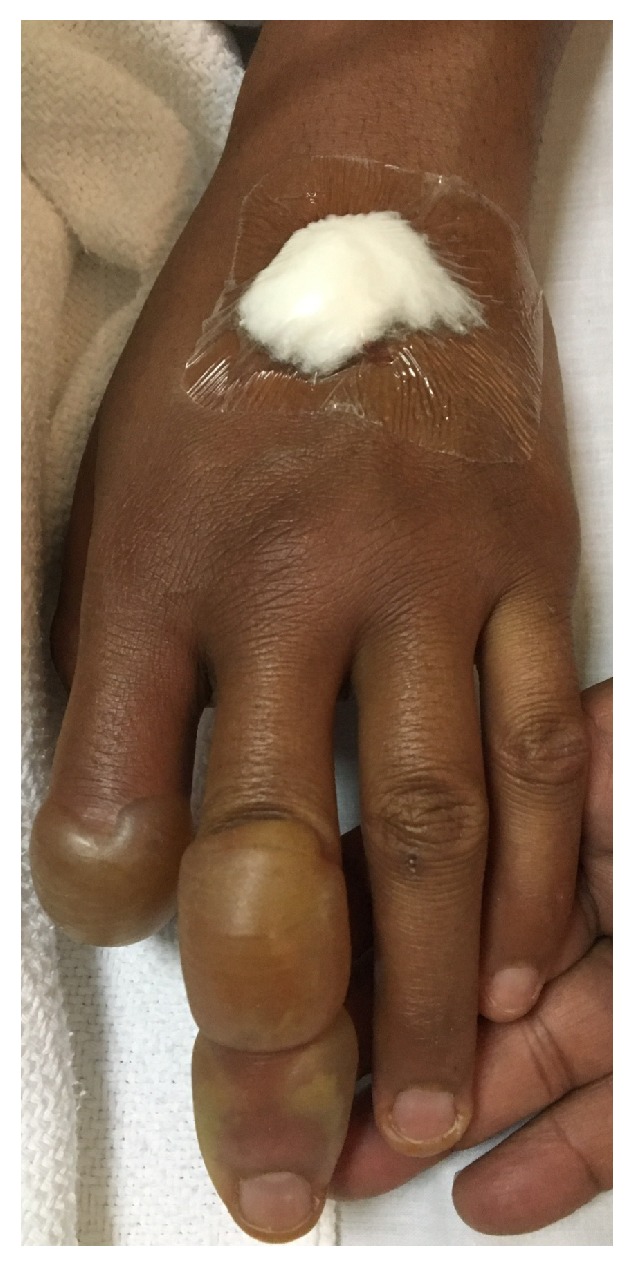
Tense blisters filled with serous fluid over dorsal hand.

**Figure 2:**
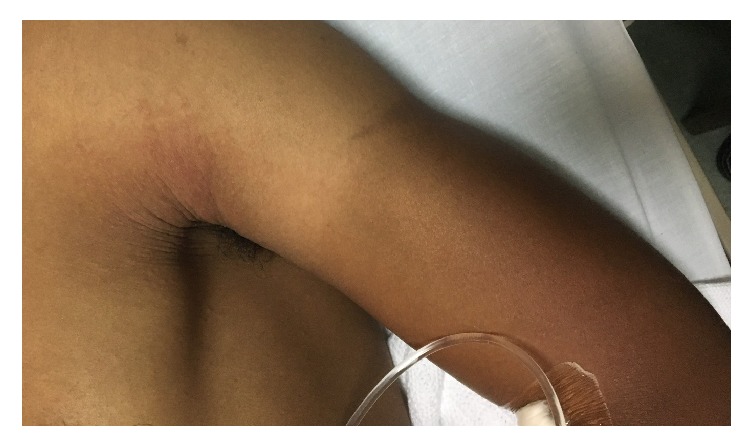
Well defined erythema over exposed surface of left arm and miliaria rubra lesions over axilla.

We report a 27-year-old man known case of schizophrenia on clozapine who developed a painful erythema with tense skin blisters coalescing to form bullae filled with serous fluid on bilateral upper limbs few hours after excessive sunlight exposure (Figure 1). The patient reported being exposed directly to the sun without sun protection when he was on vacation. He also developed miliaria rubra over bilateral axillae and back which is attributed to heat exposure (Figure 2). The patient works in the military and denies previous episodes of sunburns before starting clozapine. He was managed with pain killers and supportive treatment and totally recovered 2 weeks after the episode.

## 3. Discussion

Photosensitivity is a condition that occurs when ultraviolet radiation interacts with a medication to produce an adverse cutaneous drug eruption. Drug-induced photosensitivity reactions are divided into phototoxic reactions and photoallergic reactions. Drug-induced phototoxic reactions are the most common type of drug-induced photosensitivity. In a phototoxic reaction, ultraviolet radiation converts drug within the skin to a toxic metabolite. Phototoxic reactions occur in all individuals exposed to both high doses of medication and radiation at appropriate wavelengths. A phototoxic reaction produces an immediate exaggerated sunburn with edema and a burning sensation. The less common photoallergic reactions require an immune-mediated response. In photoallergic reactions, ultraviolet radiation causes a drug to become an antigen, which then triggers an allergic response to sunlight. Unlike phototoxic reactions, which occur only on sun-exposed skin site, photoallergy reactions can cause cutaneous changes in areas unexposed to sunlight. Clinically, patients with photoallergic reactions present with cutaneous changes that are eczematous in nature. Additionally, unlike the rapid response seen in phototoxic reactions, photoallergy reactions usually do not appear until 24 to 72 hours after exposure to the sun. Several antipsychotic medications can cause photosensitivity, most commonly the phenothiazine antipsychotics chlorpromazine and thioridazine [5].

Our patient was on clozapine, an atypical, tricyclic dibenzodiazepine antipsychotic that has multiple clinical advantages that differentiate it from typical antipsychotics. Clozapine has a relatively high affinity for the serotonin (5-HT) 2, D4, muscarinic, and alpha adrenergic receptor but weak affinity for the D2 receptor. Current evidence suggests the 5-HT2A and D4 receptor antagonist properties of clozapine, together with its weak D2 blocking properties, contribute the most to its advantages and to the reduction of extrapyramidal symptoms as well [2]. Clozapine chemical structure does not feature a prominent photosensitizing parts. However, clozapine has been described to cause a photosensitivity in 56-year-old man with paranoid schizophrenia presenting as erythema over exposed areas of the body [6]. Amisulpride, which is also a second-generation antipsychotic, has been reported to cause photosensitivity in a 38-year-old woman with paranoid schizophrenia [5]. Other atypical antipsychotics like olanzapine and aripiprazole were attributed to cause photoonycholysis in a 47-year-old female with bipolar disorder [7]. In addition, olanzapine has been described in one report to cause a pellagroid photosensitive skin rash [8]. Other cutaneous reactions reported in association with clozapine include urticaria, exanthematous reactions, serum sickness-like syndrome, sweets syndrome, erythema multiforme, and Steven-Johnson syndrome [3].

## 4. Conclusion

Photosensitivity is an underrecognized but possible side effect of second-generation antipsychotics. Further studies are needed to determine the precise mechanisms involved. Patient counseling regarding sun protection and the avoidance of excessive sunlight exposure may be necessary.
